# Novel method for the high-throughput processing of slides for the comet assay

**DOI:** 10.1038/srep07200

**Published:** 2014-11-26

**Authors:** Mahsa Karbaschi, Marcus S. Cooke

**Affiliations:** 1 Oxidative Stress Group, University of Leicester, Leicester, LE2 7LX, UK

## Abstract

Single cell gel electrophoresis (the comet assay), continues to gain popularity as a means of assessing DNA damage. However, the assay's low sample throughput and laborious sample workup procedure are limiting factors to its application. “Scoring”, or individually determining DNA damage levels in 50 cells per treatment, is time-consuming, but with the advent of high-throughput scoring, the limitation is now the ability to process significant numbers of comet slides. We have developed a novel method by which multiple slides may be manipulated, and undergo electrophoresis, in batches of 25 rather than individually and, importantly, retains the use of standard microscope comet slides, which are the assay convention. This decreases assay time by 60%, and benefits from an electrophoresis tank with a substantially smaller footprint, and more uniform orientation of gels during electrophoresis. Our high-throughput variant of the comet assay greatly increases the number of samples analysed, decreases assay time, number of individual slide manipulations, reagent requirements and risk of damage to slides. The compact nature of the electrophoresis tank is of particular benefit to laboratories where bench space is at a premium. This novel approach is a significant advance on the current comet assay procedure.

Single cell gel electrophoresis, or the comet assay, continues to attract growing interest as a tool to study the formation and repair of DNA damage, both *in vitro* and *in vivo*, as markers of genotoxicity. Furthermore, interest in the comet assay is no longer restricted to academic institutions, as there is now significant interest from industry in comet assay development and validation, for example for drug genotoxicity screening. Indeed it has been the pharmaceutical industry which has largely driven the development of Organisation for Economic Cooperation and Development guidelines for the comet assay, and it has been introduced as part of the regulation of chemicals within the European Commission's Registration, Evaluation and Authorisation of Chemicals Programme.

Although there are neutral^1^ and enzyme-modified variants of the comet assay^2^,^3^,^4^, the most widely employed variant is the alkaline comet assay (ACA), which can be used to detect and quantify strand breaks (both double and single), along with alkaline labile sites^5^. Whilst there have been some significant attempts to improve inter-laboratory agreement in levels of damage measured, largely driven by the European Comet Assay Validation Group^6^,^7^,^8^, and some new applications e.g. the assessment of DNA damage in whole blood^9^, the actual comet assay protocol has remained largely unchanged since it was originally described by Östling & Johansson^10^ and Singh et al.^11^. All variants of the comet assay involve numerous steps (Figure 1) and, with the exception of a few recent reports^12^,^13^,^14^,^15^,^16^, invariably require that microscope slides, coated with cell-containing agarose gels, are manipulated individually. These small, thin, agarose gels are delicate and at risk of damage or loss at each manipulation step, jeopardising the success of the experiment. This also makes the process time-consuming, as a typical experiment may involve up to 40 slides – a maximum determined by the time it takes to manipulate that number of slides, together with being the maximum number of slides that can be accommodated in the large electrophoresis tanks commonly used in the comet assay. The size of the electrophoresis tank is also an issue as in order to run 40 slides simultaneously, a typical tank would have a footprint of 33 × 59 cm, and is placed within a larger tray of ice, which is 60 × 75 cm, to provide cooling to the tank – and hence occupies a significant area of the bench.

On average, performing the comet assay will occupy much of three days, this excludes “scoring” of the comet assay slides to quantify the DNA damage present, which is also time-consuming. With the burgeoning development of high-throughput, or automated approaches for scoring comets, comet slide processing is clearly a bottleneck in the overall assay. However, there are no available solutions currently for improving and increasing comet slide manipulation and throughput, together with decreasing the footprint and throughput of the electrophoresis step. We have developed a method by which comet assay slides can be manipulated simultaneously in units of 25, not only does this decrease the risk of damage to the gels, it also speeds up the comet assay process. Our approach also offers the advantage of decreasing the footprint of the electrophoresis tank, through a novel design. Combined this represents a significant improvement over the conventional approach, providing a means for high throughput comet assay.

## Methods

### Materials

Simultaneous manipulation of up to 25 comet assay slides was achieved by using a polyoxymethylene rack, which was termed the high throughput (HT) rack (Figure 2A). The same rack allowed electrophoresis to be performed with the slides held, lengthwise, in a vertical orientation. A custom-made electrophoresis tank (HT Tank 1; Figure 2B) was already available within our laboratory, and proved suitable to demonstrate proof-of-principle, but required the HT rack to be shortened to fit into the tank. The tank design was then improved upon so as to accommodate two, full size HT racks (named HT Tank 2; manufactured by Cleaver Scientific Ltd, Rugby, UK), and used for all subsequent experiments involving further testing of the HT rack (Figure 2C, right).

### Effect on comet shape of performing electrophoresis on slides held vertically in the HT rack

The human keratinocyte cell line (HaCaT), which was a kind gift from Professor N.E. Fusenig (Deutsches Krebsforschungszentrum, Heidelberg, Germany^17^, was used for all ACA experiments. Cells were seeded in 12 well plates (Greiner Bio-One GmbH, Frickenhausen, Germany) and incubated overnight. After removing the medium, the cells were washed with PBS, and then exposed to a variety of concentrations of freshly prepared hydrogen peroxide (0–100 μM; Sigma-Aldrich, Dorset, UK) for 30 min on ice. After exposure, the H_2_O_2_ was removed by washing with PBS, prior to analysis by conventional and our novel HT ACA. The ACA method was essentially as described previously^18^. Briefly, 80 μL of low melting point agarose gel (Invitrogen, Paisley, UK; containing approximately 1.2 × 10^4^ cells) were dispensed onto glass microscope slides, coated previously with 1% normal melting point agarose. The agarose was allowed to set, under a 22 × 22 mm cover slip (VWR International, distributed by Fisher Scientific, Loughborough, UK) by placing the slides on ice. The cover slips were then removed and the slides either processed individually, according to conventional ACA, or simultaneously when placed vertically in an HT rack (six slides were used per experiment, two slides per treatment condition, and the spare spaces in the HT rack were filled with ‘blank' slides i.e. slides without gels). The individual slides or slides in the HT rack were then left overnight in ice-cold lysis buffer (100 mM disodium EDTA, 2:5 M NaCl, 10 mM Tris-HCl, pH 10, containing 1% triton X-100 which was added freshly). In the case of the HT rack this step, and all steps involving washing/neutralisation/draining/drying/rehydration/staining etc, was performed in a rack staining dish (Figure 2A). The individual slides or slides in the HT rack were then placed in ice cold water for 30 min. Afterwards, the individually manipulated slides were laid flat, in a horizontal orientation, in the HT Tank 1 together with the second set of slides, which were place vertically in the same tank, using the HT rack. All slides were covered with cold alkaline electrophoresis buffer (300 mM NaOH, 1 mM disodium EDTA, pH ≥ 13) for 20 min and then electrophoresis performed at 27 V and 300 mA for 20 min (0.9 V/cm). Neutralisation was then performed using 0.4 M Tris-HCl, pH 7.5 for 20 min prior to washing with distilled water then the slides allowed to dry. All procedures were carried out under subdued light to minimise possible adventitious DNA damage. For staining, the slides in the HT rack were submerged in distilled water to re-hydrate the slides prior to being submerged in freshly made solution of 2.5 μg/mL propidium iodide for 20 min. The slides were washed again for 30 min and allowed to drain and dry whilst still in the rack. In contrast, the other slides were each individually manipulated for the rehydration, staining, washing and drying steps. All slides were then observed and scored by fluorescent microscopy (50 cells per gel; 100 cells per treatment), and percentage tail DNA of the comets was recorded, using comet assay IV analysis software, version 4.2 (Perceptive Instruments, Haverhill, Suffolk, UK). These experiments were repeated in their entirety on three different occasions.

### Effect of buffer volume on tank voltage/current parameters

With proof-of-principle established using HT Tank 1, HT Tank 2 was used for all subsequent experiments. The size/shape and the presence of additional slides in the HT Tank 2 altered the buffer volume required to cover the slides. Differences in buffer volume, compared to those used in conventional ACA, were investigated in terms of the effect on voltage and/or current. The effect of the optimal buffer conditions was then tested on comet assay electrophoresis (below).

### Concentration-response and repeatability of the HT comet assay

The effect of the optimised materials and assay conditions was then tested to study their effect on electrophoresis of comets. This was examined by testing the ability to detect a concentration-response, together with a study of repeatability and comparison with conventional ACA. HaCaTs were again exposed to a variety of concentrations of hydrogen peroxide (0–100 μM) prior to analysis by the novel HT ACA and conventional ACA, as described above, with the inclusion of the optimised buffer/current/voltage conditions.

### Statistical analysis

Differences between treatments were assessed by analysis of variance, using a Kruskal-Wallis and Dunn's Multiple Comparison Test. Statistical analyses were performed using GraphPad Prism, version 6.02 (GraphPad, CA, USA).

## Results

### Effect on comet shape of performing electrophoresis on slides held vertically in the HT rack

**Figure 1:**
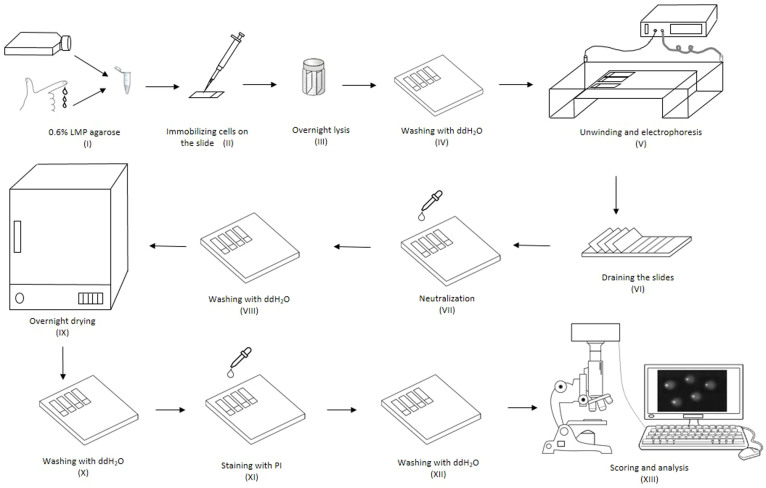
Overview of the typical alkaline comet assay procedure. (i) A single cell suspension of the cells under investigation is mixed with low melting point agarose. (ii) The cell/agarose mix is layered onto glass microscope slides, pre-coated with agarose, and the agarose allowed to set. (iii) The cells are lysed under high pH before (iv) washing with pure water. The presence of strand breaks and high pH allows the cellular DNA to unwind. (v) Electrophoresis draws the DNA out of the nucleoid body forming a ‘tail'. The amount of migration (the amount of DNA in the tail versus the head) is proportional to the initial amount of DNA damage. The slides are then (vi) drained, (vii) neutralised and (viii) washed with pure water before (ix) drying overnight. Following further (x) washing in pure water, the slides are (xi) stained, (xii) washed and finally (xiii) scored and analysed, typically using fluorescent microscopy and image analysis software.

In order to assess the effect of performing electrophoresis on slides in the vertical orientation in the HT rack, the level of DNA damage and the quality of comets were compared with performing ACA in the conventional, horizontal orientation. The results showed that the orientation and the shape of the comets which were run vertically in the HT rack (Figure 3A) were identical to those run horizontally (Figure 3B). Furthermore the data obtained after scoring the comets indicated that there was no significant difference in percentage tail DNA between the samples run horizontally or vertically (P > 0.05; Figure 3C). Additionally, using the HT racks provided a 60% decrease in time spent manipulating slides (i.e. Figure 1, steps III, IV, V, VI, VII, VIII, IX, X, XI, XII), compared to conventional ACA, together with decreasing the risk of damage to gels during manipulation.

### Effect of buffer volume on tank voltage/current parameters

All subsequent experiments were performed with the HT Tank 2. However, it was first necessary to find a minimal buffer volume, which covered the HT racks, and achieved voltage/current conditions closest to those used in conventional ACA. We immediately identified that the power supply used normally for electrophoresis would not suffice, (Power Pac 300, Bio-Rad) as it had difficulty achieving a current larger than 400 mA required to reach 27 V, and a power supply with a greater current range was required. The CS-330V power supply (CS-300V; Cleaver Scientific Ltd, Rugby, UK) proved to be perfectly suited to this application.

A number of combinations were attempted, and the optimal buffer volume for HT Tank 2 was determined to be 550 mL, which gave 27 V and 450 mA (Table 1). These conditions were therefore used in subsequent experiments.

### Concentration-response and repeatability of the HT ACA

The results in Figure 4 show the ability of the HT ACA to sensitively and reproducibly quantify H_2_O_2_-induced DNA damage. The HT Tank 2 can accommodate two HT racks, in two “zones”, one closer to the anode, and one closer to the cathode. Crucially, no field effects, or heterogeneity in the electrophoretic field were detected which would have been manifested as significant differences in response in zone 1 versus zone 2 (P > 0.05; Figure 4). Furthermore, the levels of intra- and inter-experiment variability appeared to be no different to those seen with conventional ACA electrophoresis.

The use of the HT racks, by eliminating the need to manipulate individual slides, significantly decreased the processing time for the lysis, electrophoresis, neutralisation and staining steps, together with all of the associated wash steps (Figure 1, steps iii to xii). Advantageously, as a result of indirect manipulation of the slides, the fragile gels were less likely to be damaged during the comet assay steps.

## Discussion

There are two major limitations to the throughput of the comet assay. The first is the scoring of comets – typically this involves manually determining the level of DNA damage in fifty cells per gel and two gels per treatment, within a single experiment. To address this, there has been an emergence of automated image acquisition and analysis platforms, such as that reported by Ritter and Knebel^19^. The second limitation relates to sample work up. As evident from Figure 1, the gel-coated microscope slides undergo numerous manipulations during the assay procedure. Each of these possesses the potential for the fragile gels to be lost or damaged, risking the entire experiment. There have been a number of approaches to increase the throughput of the comet assay at the sample work up stage^12^,^13^,^14^,^15^,^16^, but in all of these have represented a departure from the conventional use of microscope slides to support the cell-containing gels, and therefore significant changes in procedure for the laboratories that undertake this assay. Observations from a recent study have indicated that changing a well-established comet assay procedure can be problematic for some laboratories^7^, and would therefore be best avoided. Furthermore, a departure from the use of microscope slides may also make more difficult to perform certain variants of the comet assay, such as the enzyme-modified ACA.

**Figure 2:**
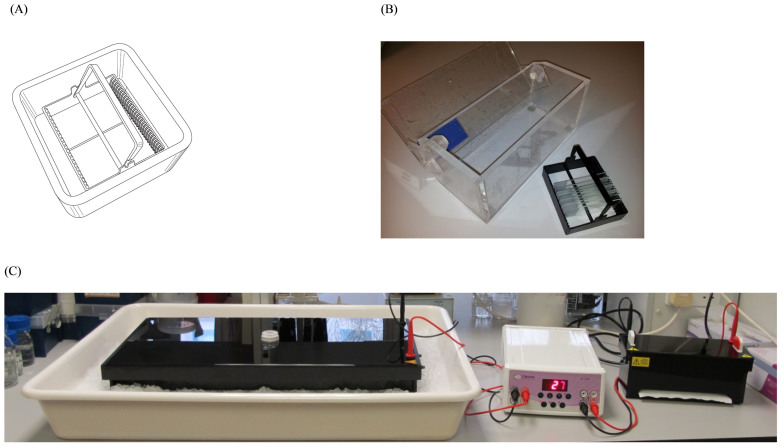
(A) Representative illustration of the HT racks, which can accommodate up to 25 slides, and the staining dishes in which lysis, neutralisation, staining and all associated wash steps are performed. (Figure 2A is reproduced here with the kind permission of Comery, Hill & C°., Benthall, UK). (B) The HT Tank 1 and HT rack. The HT Tank 1 was used in preliminary, proof-of-principle experiments. (C) Demonstration of the size difference between the conventional ACA apparatus (left) and the HT Tank 2 (right), which are separated by a power supply.

**Figure 3:**
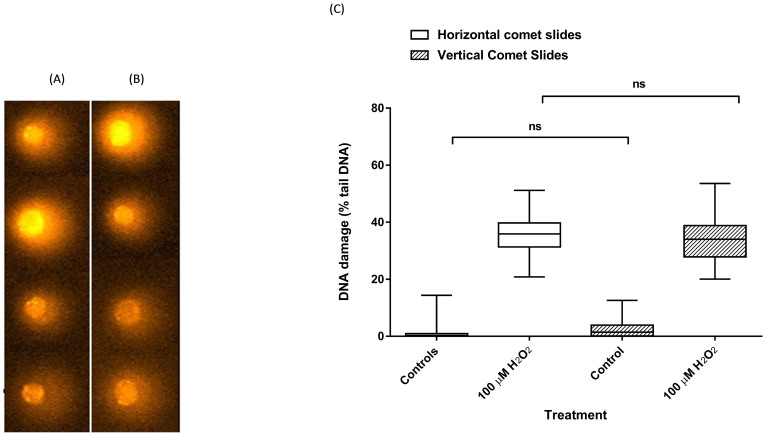
The effect of comet slide orientation during electrophoresis on comet appearance and quality. HaCaTs were incubated with 100 μM H_2_O_2_ prior to analysis by conventional alkaline comet assay, or the new method using the HT rack. Representative images of comets following electrophoresis performed in the same electrophoresis tank with the comet slides held (A) vertically in a HT rack, and (B) horizontally, as is the convention. (C) Quantification of H_2_O_2_-induced DNA damage in HaCaTs determined by ACA with electrophoresis performed in either the horizontal or vertical orientation. Error bars represent the median and max/min of 200 individual determinations from two independent experiments (ns = not significant).

**Figure 4:**
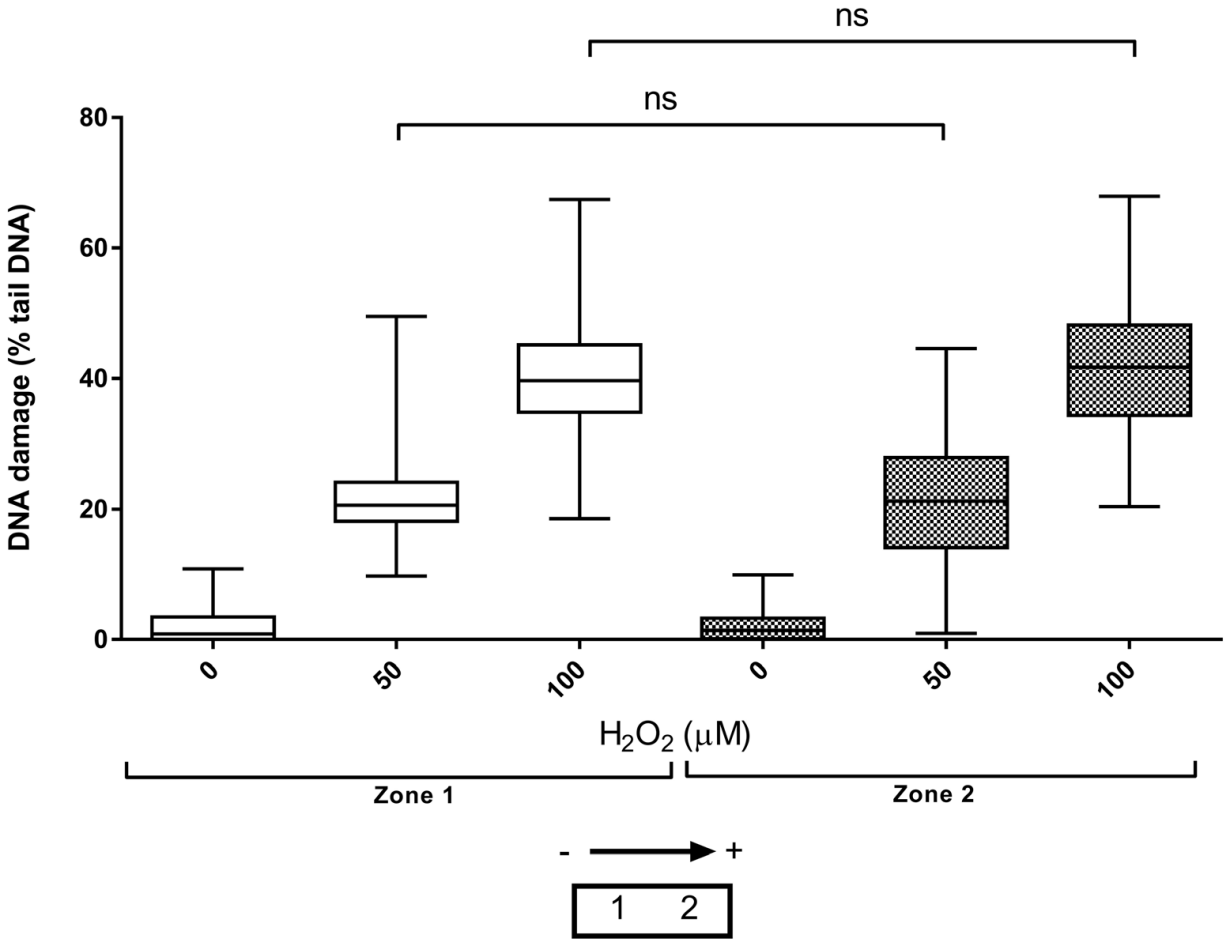
Concentration-response and repeatability of the HT ACA, using the HT Tank 2. The experiments with 0 and 100 μM H_2_O_2_ were performed three times, and those with 50 μM H_2_O_2_, twice, with the error bars representing the median and max/min of 300 and 200 individual determinations, respectively. The HT Tank 2 has two “zones” in which an HT rack can be placed, which has no effect on the results (ns = not significant). The lower figure indicates the location of the two “zones” within the tank when viewed from above. Voltage ran from left to right (anode to cathode).

We report a novel improvement to the comet assay, demonstrated using the ACA, but which could be applied to all variants. We discovered that electrophoresis could be performed successfully (i.e. the shape and size of the comets are unaffected) with the slides held in a vertical orientation, rather than horizontally, as is the convention. As it was only the orientation of the slides that had been altered, the duration of electrophoresis did not need to be increased to achieve identical results to the existing ACA, unlike other high throughput methods^15^. This change of orientation brought a number of improvements to the assay. Multiple slides can be held in a rack, allowing their simultaneous manipulation, in the present case 25 at a time, which not only makes the assay procedure easier, so less skill is required, but also speeds up the process as the slides can remain in the racks throughout all of the comet assay steps. This also provides protection to the slides and minimises the risk of damage to the delicate gels adhered to the slides. With the slides in the vertical orientation, they also occupy less space, so the HT electrophoresis tank has a smaller footprint than conventional tanks (210 cm^2^ vs. 1,947 cm^2^), and in its present format (HT Tank 2; Figure 2C), it has built-in cooling, obviating the need for an external tray of ice, whose additional space requirement would take the total footprint for a single tank to 3,420 cm^2^. This offers the ability to process over six times as many slides as a horizontal tank of the same proportions (excluding the required ice tray), and keeps all the slides in a more uniform orientation with respect to the electrophoretic field.

The HT tank also requires smaller buffer volumes, with accompanying cost savings. To further aid throughput, multiple tanks can be run simultaneously, from a single power supply, significantly increasing the number of slides run, with minimal increases in bench space requirement. Taken together, this novel high throughput approach represents a significant advantage over the existing comet assay procedure, whilst retaining key components of the conventional assay.

## Figures and Tables

**Table 1:** Effect of buffer volume on voltage/current parameters using a Cleaver scientific Powerpac (CS-300V) in conjunction with the HT Tank 2

Volume (mL)	Amp (mA)	Voltage (V)	Result
700	300	Fluctuated between 17 and 18	OK
700	400	Fluctuated between 20,21 and 22	OK
700	500	26	OK
700	530	27	OK
600	300	Fluctuated between 21 and 22	OK
600	350	Fluctuated between 23 and 24	OK
600	399–400	26	OK
600	425	26	Took 2 min to reach 425 mA and 27 V, then OK
600	430	26	OK
600	440	26	OK
600	450	Fluctuated between 26 and 27	Took 3 min to reach 450 mA and 27 V, then OK
600	460	27	Took 3 min to reach 460 mA and 27 V, then OK
550	350	22	OK
550	400	25	OK
550	420	26	OK
550	440	Fluctuated between 26 and 27	OK
550	450	27	OK

## Notes

Publication of this article was funded in part by Florida International University Open Access Publishing Fund.
